# Dr. Guy Bryan Miller

**Published:** 1903-06

**Authors:** 


Dr. Guy Bryan Miller, who died at Paris, France, April 7, 1903, was born in New Rochelle, February 23, 1872, and was the son of Charles Griffin Miller. He was graduated from Yale University in 1894, and from the College of Physicians and Surgeons, Medical Department of Columbia University, in 1898. He served two years in St. Luke's Hospital, New York, and studied another two years in Vienna. From there he went to Paris in 1892.

Dr. Miller was well known in Buffalo. The funeral was held from the house of Mr. Frank Hamlin on North Street, the Rev. H. B. Master officiating. The burial was at Forest Lawn.

